
JOURNAL INFORMATION
==============================
NLM Title Abbreviation: Wirtschaftsdienst
Journal ID: Wirtschaftsdienst
NLM Journal ID: 101086612

Wirtschaftsdienst (Hamburg, Germany : 1949)

ISSN: 0043-6275

ARTICLE INFORMATION
==============================
PMCID: PMC7368620
PMID: 32834162
DOI: 10.1007/s10273-020-2686-3
Article ID: 2686
Article version: 1
Subjects: Kurz Kommentiert

Das Ende des Teilens?

Car sharing in the crisis: The end of sharing?

Kolleck Aaron akolleck@inet.tu-berlin.de

Stocker Volker vstocker@inet.tu-berlin.de

grid.6734.6 0000 0001 2292 8254 Weizenbaum-Institut für die vernetzte Gesellschaft, TU Berlin, Hardenbergstraße 32, 10623 Berlin, Deutschland
Electronic publication date: 2020 Jul 18
Print publication date: 2020
Volume: 100
Issue: 7
First page: 478
Last page: 478
Copyright: © Der/die Autor(en) 2020
License: Open Access Dieser Artikel wird unter der Creative Commons Namensnennung 4.0 International Lizenz veröffentlicht, welche die Nutzung, Vervielfältigung, Bearbeitung, Verbreitung und Wiedergabe in jeglichem Medium und Format erlaubt, sofern Sie den/die ursprünglichen Autor(en) und die Quelle ordnungsgemäß nennen, einen Link zur Creative Commons Lizenz beifügen und angeben, ob Änderungen vorgenommen wurden.
Die in diesem Artikel enthaltenen Bilder und sonstiges Drittmaterial unterliegen ebenfalls der genannten Creative Commons Lizenz, sofern sich aus der Abbildungslegende nichts anderes ergibt. Sofern das betreffende Material nicht unter der genannten Creative Commons Lizenz steht und die betreffende Handlung nicht nach gesetzlichen Vorschriften erlaubt ist, ist für die oben aufgeführten Weiterverwendungen des Materials die Einwilligung des jeweiligen Rechteinhabers einzuholen.
Weitere Details zur Lizenz entnehmen Sie bitte der Lizenzinformation auf http://creativecommons.org/licenses/by/4.0/deed.de.
License URL: https://creativecommons.org/licenses/by/4.0/

issue-copyright-statement: © The Author(s) 2020

==============================
DOI: 10.1007/s10273-020-2686-3

Carsharing in der Krise

### Das Ende des Teilens?

Carsharing hat sich hierzulande als ernstzunehmende Alternative für das eigene Auto und zu einem prägenden Element der Mobilitätslandschaft entwickelt. Laut Bundesverband CarSharing gibt es seit Beginn des Jahres 2020 in Deutschland rund 25.000 geteilte Pkw, die ungefähr zu gleichen Teilen auf stationsgebundene und -ungebundene Angebote entfallen. Obgleich in der Sharing Economy das Mantra „teilen statt besitzen" den temporären und flexiblen Zugang zu oft teuren Ressourcen verspricht und so Teilhabe auch ohne exklusives Privateigentum ermöglicht, wird dieses Mantra in der aktuellen Krise zum Problem.

Ein zentrales Versprechen des Carsharings ist dessen Beitrag zu einer effizienteren und nachhaltigeren Mobilität und damit verbundene individuelle und gesellschaftliche Vorteile. Auf individueller Ebene ermöglicht Carsharing in erster Linie Teilhabe, d.h. die Nutzung eines Fahrzeugs, ohne die Kosten und Risiken der Anschaffung und Haltung eines Autos tragen zu müssen. Gesamtgesellschaftlich betrachtet kann die geteilte Nutzung von Fahrzeugen die Zahl der Fahrzeuge, die für die Bedienung der Mobilitätsnachfrage erforderlich ist, verringern. So können unter anderem (externe) Kosten im Zusammenhang mit der Produktion reduziert sowie notorisch knappe Parkkapazitäten entlastet werden. Für die Beurteilung der Vorteilhaftigkeit ist eine zentrale Frage zu beantworten: Stellt Carsharing tatsächlich ein Substitut für privaten Autobesitz dar?

Die Literatur liefert uneinheitliche Antworten. Es zeigt sich, dass die Unterscheidung zwischen stationsgebundenem und -ungebundenem Carsharing von wesentlicher Bedeutung ist. Eine Mehrheit der verfügbaren Studien deutet auf einen substitutiven Zusammenhang zwischen stationsgebundenem Carsharing und privatem Autobesitz hin. Im Gegensatz dazu sind die Ergebnisse bei ungebundenem Carsharing widersprüchlicher: Einzelne Studien legen eine Substitution nahe, andere finden keinen oder sogar einen negativen und somit komplementären Zusammenhang. Ursachen der Unterschiede in der entsprechenden Substitutionswirkung werden in der Nutzung der geteilten Fahrzeuge vermutet: stationsgebundenes Carsharing kommt in seiner Funktionalität näher an ein privates Auto heran, da diese vor allem für längere Fahrten genutzt werden, z. B. für Einkäufe oder Kurzurlaube. Stationsungebundenes Carsharing stellt im Wesentlichen ein Angebot der innerstädtischen Mobilität dar. Dort bedient es eine Nachfrage, die zwischen Taxi/Uber und öffentlichem Nahverkehr zu verorten ist.

In Anbetracht der aktuellen Krise stellt sich nun die Frage, ob bzw. wie sich die Einstellung zum Teilen von Fahrzeugen grundsätzlich und nachhaltig ändert. Dabei sind mehrere Faktoren zu berücksichtigen. Exogene Faktoren wie die in Deutschland temporär eingeführten Maßnahmen zur Eindämmung des Coronavirus beeinflussen das Mobilitätsverhalten und haben zu einer dramatisch sinkenden Mobilitätsnachfrage geführt. Krisenbedingte Beschränkungen haben das Potenzial kognitiv neues oder verändertes Verhalten zu etablieren sowie organisatorische Veränderungen und Innovationen herbeizuführen. Als Beispiele dienen die Verbreitung von Home-Office und Online-Lehre, sowie die Verlagerung der Präferenzen hin zu alternativen Verkehrsmitteln. Während entsprechende Anpassungen teilweise transitorischer Natur sein dürften, bleibt abzuwarten, wie nachhaltig sie Verhalten im Zustand des „new normal" verändern werden.

Es fällt auf, dass individuelle Mobilität – z. B. per Auto, Fahrrad oder Motoroller – in der Krise an Attraktivität gewinnt. Geteilte Mobilität wird aufgrund der (wahrgenommenen) Gefahr einer Ansteckung eher vermieden. Dies belegen unter anderem die von Google und Apple Maps veröffentlichten Mobilitätsberichte. Im Unterschied zu geteilten Verkehrsmitteln (z. B. öffentliche Verkehrsmittel, Ride-Pooling oder Ride Hailing) kann bei Carsharing der persönliche Kontakt vermieden werden. Dennoch ist der Grad an Kontrolle nicht so hoch wie bei einem privaten Pkw. Gegen einen privaten Pkw und Eigentum im Allgemeinen sprechen besonders in der Krise sinkende Einkünfte und eine höhere Unsicherheit über zukünftige Einnahmen. Carsharing als Substitut zum privaten Autobesitz könnte so an Attraktivität gewinnen. Anbieter könnten zumindest so lange profitieren, wie es dadurch eine Nachfrage nach individueller, aber unregelmäßiger Mobilität gibt.

Nichtsdestotrotz wird die Krise bestehende Konsolidierungstendenzen im Carsharing-Sektor vermutlich verstärken. Dabei bleibt offen, ob sich nur die Geschwindigkeit oder auch der Kurs der strukturellen Anpassungsprozesse grundsätzlich ändert. Der Ruf nach finanzieller Unterstützung durch den Staat wurde bereits laut.

Aaron Kolleck & Volker Stocker
TU Berlin /Weizenbaum-Institut für die vernetzte Gesellschaft
akolleck@inet.tu-berlin.de

© Der/die Autor(en) 2020. Open Access: Dieser Artikel wird unter der Creative Commons Namensnennung 4.0 International Lizenz (https://creativecommons.org/licenses/by/4.0/deed.de) veröffentlicht.

Open Access wird durch die ZBW – Leibniz-Informationszentrum Wirtschaft gefördert.
